# Enhancement of Piezoelectric Performance in PVDF via ZnO Doping and Its Application in Wearable Real-Time Monitoring of Human Radial Pulse

**DOI:** 10.3390/bios16040187

**Published:** 2026-03-24

**Authors:** Hao Zhu, Xiang Guo, Qiang Liu, Qian Zhang

**Affiliations:** 1 School of Integrated Circuit Science and Engineering, University of Electronic Science and Technology of China, Chengdu 611731, China; haozhu@jsatec.com; 2 Mianyang Municipal Health Commission, Mianyang 621000, China; swjw301@163.com; 3 School of Materials and Energy, University of Electronic Science and Technology of China, Chengdu 611731, China; qiangrye@163.com

**Keywords:** piezoelectric, PVDF-TrFE, ZnO, heart rate monitoring

## Abstract

Flexible piezoelectric materials demonstrate broad application potential in wearable health monitoring, human–machine interaction, and biosensing. However, the piezoelectric response of pure PVDF-TrFE is limited and insufficient to meet the requirements for highly sensitive sensing. In this study, ZnO/PVDF-TrFE composite films with varying ZnO doping contents (3–11 wt%) were fabricated and systematically characterized in terms of their structural, thermal, and electrical properties. The results indicate that ZnO significantly promotes the formation of the polar β-phase in PVDF-TrFE, with the maximum β-phase content (*F_β_* = 24.76%) and optimal piezoelectric performance achieved at 9 wt% ZnO doping. Devices based on this optimal composition exhibited stable ultrasonic transmission and reception capabilities under high-frequency pulse excitation, enabling sensitive detection of minor static pressure variations (e.g., contact pressure) through changes in ultrasonic echo signals, thereby realizing wearable conformity monitoring. Moreover, a sensor designed with a three-channel flexible substrate successfully captured human wrist pulse signals with high accuracy, demonstrating the practical utility and reliability of the device in flexible bio-electronic sensing applications.

## 1. Introduction

Flexible piezoelectric materials demonstrate broad application prospects in fields such as wearable health monitoring, human–machine interaction, and biosensing [1,2,3,4,5]. Among them, poly(vinylidene fluoride-trifluoroethylene) (PVDF-TrFE) has attracted significant research attention owing to its intrinsic piezoelectricity, flexibility, and biocompatibility [6,7,8]. However, the piezoelectric response of pure PVDF-TrFE is limited, making it difficult to meet the requirements of highly sensitive sensing [9]. To address this, researchers often introduce nanofillers to form composite materials, thereby enhancing their piezoelectric and dielectric properties. For example, the addition of carbon nanotubes (CNTs: carbon nanotubes) or graphene can effectively improve the electrical conductivity and mechanical strength of the composites [10,11,12], while incorporating ceramic fillers such as barium titanate (BaTiO_3_) can significantly increase the dielectric constant [13,14,15]. Among these fillers, zinc oxide (ZnO) has garnered considerable interest due to its distinctive advantages [16,17]. It is a multifunctional nanomaterial that exhibits both piezoelectric and semiconducting properties. The incorporation of ZnO not only effectively enhances the piezoelectric output and dielectric performance of composites but also leverages its excellent biocompatibility and relatively low production cost, offering a promising solution for developing high-performance, low-power-consumption, and practically viable electronic skin sensors.

Despite the considerable attention given to ZnO/PVDF-TrFE composites, several key challenges remain in current research: (1) due to the influence of preparation processes, the quantitative relationship between ZnO doping content and the material’s crystal structure, particularly the β-phase content, is not well-defined [7,18]. The doping concentration of ZnO has a substantial impact on the piezoelectric properties of PVDF-TrFE composites, and a consensus in the literature indicates that the optimal doping range is typically between 4 and 10 wt%. Kumar et al. [19] fabricated composite nanofibers via electrospinning and also confirmed that the voltage output peaked at a ZnO content of 7%, and decreased beyond this level due to agglomeration effects. Han et al. [20] found that as the ZnO content increased from 0% to 7.5%, the piezoelectric output gradually improved and reached its optimum at 7.5%. However, further increasing the content to 12.5% led to a decline in performance due to nanoparticle agglomeration. When the ZnO doping concentration is excessively low, the scarcity of nucleation sites hinders the effective transformation of PVDF molecular chains from the non-polar α-phase to the electroactive β-phase, resulting in a limited increase in β-phase content and consequently a marginal improvement in piezoelectric performance [7]. In contrast, an excessively high doping concentration induces nanoparticle agglomeration, which not only fails to promote β-phase nucleation but also restricts polymer chain mobility and weakens interfacial interactions between the ZnO fillers and the PVDF-TrFE matrix, ultimately leading to a deterioration in piezoelectric properties [19,20]. (2) the systematic influence of ZnO content on the piezoelectric output, ultrasonic response, and dynamic sensing performance of the composites has not been fully elucidated [19,21]. (3) existing devices still exhibit limitations in flexibility, conformal stability, and accuracy in biosignal detection, which restricts their reliability in practical wearable applications [22,23]. Most existing studies focus on optimizing a single performance metric (e.g., piezoelectric coefficient d_33_) or rely solely on qualitative morphological and structural characterization, lacking a holistic and systematic investigation that spans crystallization behavior, thermal properties, electrical output, ultrasonic response, and biosignal detection [19,24,25]. Furthermore, most sensors adopt a single-channel design, which is prone to signal loss caused by positional misalignment or electrode failure in dynamic wearing scenarios, and fails to fully utilize non-destructive detection methods such as ultrasonic echoes for real-time monitoring of the device-tissue interface state.

To address these issues, we fabricated a series of ZnO/PVDF-TrFE composite films with varying ZnO doping contents (3–11 wt%). Multi-scale characterization techniques including XRD, FTIR, and DSC were employed to establish quantitative relationships among ZnO content, β-phase content, crystallinity, and thermal properties. We also constructed electrical and ultrasonic testing platforms to systematically evaluate piezoelectric output, high-frequency response, and static pressure sensing performance. A three-channel flexible sensor array was designed, which integrates ultrasonic echo signals to enable self-monitoring of device conformity and was validated for accuracy and stability in capturing human pulse signals. XRD and FTIR analyses demonstrated that the incorporation of ZnO significantly promotes the formation of the polar β-phase in PVDF-TrFE. At a ZnO content of 9 wt%, the β-phase content (*F_β_*) reached a maximum of 24.76%, corresponding to optimal piezoelectric performance. Devices based on the optimized 9 wt% formulation exhibited stable ultrasonic transmission and reception capabilities under high-frequency pulse excitation, providing a viable approach for wearable conformity monitoring and accurately capturing human wrist pulse signals.

## 2. Materials and Methods

### 2.1. Materials

PVDF-TrFE powder (Mw ≈ 520,000) was supplied by Akema Piezotech (Paris, France). ZnO powder with a particle size of 300 nm was purchased from Hebei Badu Metal Materials Co., Ltd (Baoding, China). The butanone was purchased from Chengdu Cologne Chemical Co., Ltd (Chengdu, China). ITO (indium tin oxide) conductive glass was purchased from Luoyang Guluo Glass Co., Ltd (Luoyang, China). The flexible printed circuit (FPC) boards were custom-designed and fabricated by Shenzhen Jialichuang Technology Co., Ltd (Shenzhen, China).

### 2.2. Fabrication of ZnO/PVDF-TrFE Composites

**Figure 1:**
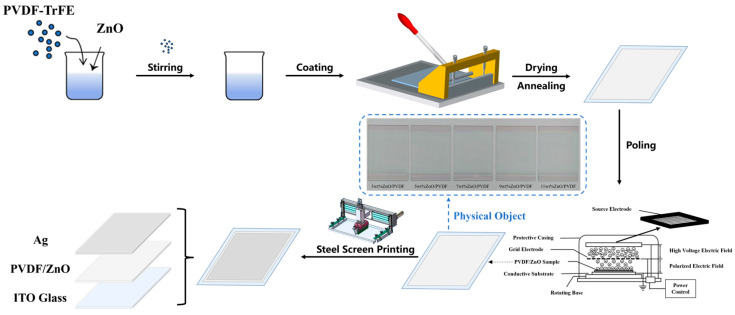
Fabrication process of ZnO/PVDF-TrFE sensors.

PVDF-TrFE powder and ZnO powder are completely dissolved in butanone solvent via magnetic stirring at varying mass ratios (97:3, 95:5, 93:7, 91:9, and 89:11 wt%) to form a homogeneous solution. The solution is then coated onto either an ITO conductive glass or an FPC flexible conductive substrate using a slot-die coating process (coating speed: 50 mm/s; doctor blade height: 220 μm), followed by drying in a vacuum oven (Shanghai Yiheng Scientific Instrument Co., Ltd., Shanghai, China) (vacuum degree: 10 Pa; time: 10 min) to obtain a dry film with a specified thickness of 20 μm. The film is annealed at 140 °C for 40 min and subsequently subjected to in situ poling (gate voltage: 7500 V; source voltage: 3500 V) to induce piezoelectric properties. A silver (Ag) layer is screen-printed onto the surface of the PVDF-TrFE film as the top electrode, while the ITO conductive glass or FPC flexible substrate itself functions as the bottom electrode. The fabricated current sensor is shown in Figure 1. Figure 1 presents schematic and photographic images of the ZnO/PVDF-TrFE composite films with different mass ratios.

### 2.3. Characterization

Scanning electron microscopy (SEM) images and energy-dispersive spectroscopy (EDS) analysis were obtained using a ZEISS GeminiSEM 300 system (Carl Zeiss, Oberkochen, Germany) (magnification: 5 K–200 K). Crystallization behavior was characterized by X-ray diffraction (XRD) using a Rigaku Ultima IV diffractometer (Rigaku, Tokyo, Japan) (scanning angle: 5–90°; scanning speed: 2°/min). Thermal analysis curves were measured with a Netzsch DSC 200 F3 (Netzsch, Selb, Germany) differential scanning calorimeter (Germany) at a heating/cooling rate of 10 °C/min. Fourier transform infrared (FTIR) spectra were collected using a Thermo Scientific Nicolet iN 10 infrared microscope (Thermo Fisher Scientific, Waltham, MA, USA) (wavenumber range: 400–4000 cm^−1^). Piezoelectric signals were recorded with an NI 9234 voltage acquisition card (National Instruments, Austin, TX, USA). Ultrasonic echo signals were acquired via a CTS-8077PR pulse transceiver (Guangdong Shantou Ultrasonic Electronics Co., Ltd., Shantou, China) and an MDO4024C oscilloscope (Tektronix, Beaverton, OR, USA).

## 3. Results and Discussion

### 3.1. Characterization of the ZnO/PVDF-TrFE Composite Films

**Figure 2:**
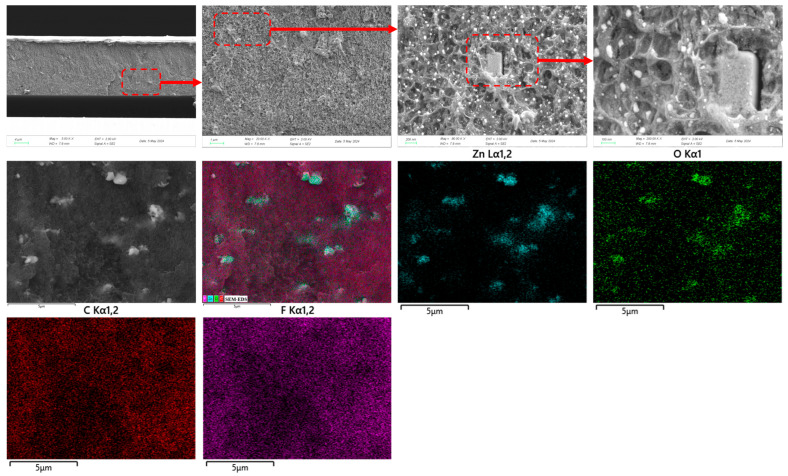
SEM and ESM-EDS images of ZnO/PVDF-TrFE composite film.

Figure 2 presents the SEM-EDS characterization results of the ZnO/PVDF-TrFE composite film. The cross-sectional SEM image of the fracture surface reveals a relatively smooth and uniform morphology with no obvious structural defects, indicating good consistency in the preparation process. At higher magnification, ZnO nanoparticles are observed to be homogeneously dispersed within the PVDF-TrFE matrix, although some localized agglomeration is evident, likely due to van der Waals forces between nanoparticles. Under ultra-high resolution, subtle structural phase transitions can be detected within the agglomerated regions, which may be associated with localized stress or alterations in crystallization behavior at the ZnO-polymer interface. As shown in Figure 2, SEM-EDS elemental mapping of the cross-section provides distribution patterns of C, O, F, and Zn. The elemental maps clearly demonstrate the continuous and uniform distribution of ZnO fillers in the PVDF-TrFE matrix. No significant elemental segregation or structural discontinuity is observed at the interface between the two phases, indicating good compatibility and strong interfacial bonding. This structural foundation supports synergistic responses of the composite under electric or mechanical stimuli.

**Figure 3:**
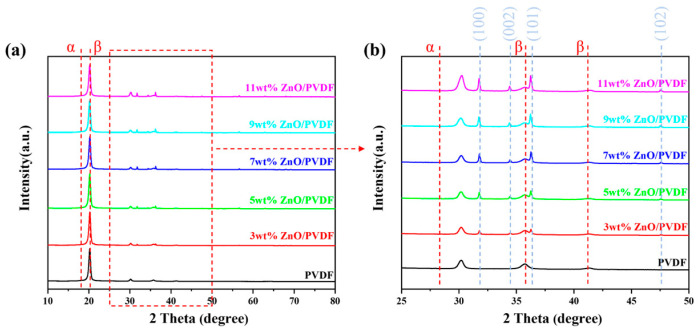
XRD patterns of ZnO/PVDF-TrFE composite films. (**a**) Full pattern in the 2θ range of 10°–80°; (**b**) magnified view in the 2θ range of 25°–50°.

Figure 3 shows the XRD patterns of ZnO/PVDF-TrFE composite films with different ZnO doping contents. The effect of varying ZnO doping levels on the crystallization behavior of ZnO/PVDF-TrFE composite films was analyzed through X-ray diffraction (XRD). In the XRD patterns, the peaks at 18.3° and 26.7° correspond to the (020) and (021) planes of the α-phase in PVDF-TrFE, respectively [26]. After the introduction of nano-ZnO powder, the characteristic α-phase peak at 26.7° disappeared, while the diffraction intensity at 18.3° decreased. The peaks located at 20.3°, 35.8°, and 41.2° are attributed to the (110)/(200), (001), and (111)/(201) planes of the β-phase crystals in PVDF-TrFE, respectively [27]. The intensities of the peaks at 20.3° and 35.8° increased with higher ZnO content, whereas the intensity of the peak at 41.2° remained unchanged. These results indicate that the β-phase crystallinity enhances with rising ZnO content in the ZnO/PVDF-TrFE composite films. This trend suggests that the incorporation of nano-ZnO effectively promotes the conformational transition of PVDF molecular chains from the α-phase to the β-phase. Such a crystal-structural transformation mechanism plays a significant role in enhancing the piezoelectric response of the material. The characteristic peaks observed at 2θ = 31.8°, 34.4°, 36.3°, and 47.5° correspond to the (100), (002), (101), and (102) crystal planes of ZnO, respectively [28,29]. The sharp diffraction peaks indicate good crystallinity of the ZnO/PVDF-TrFE composite films. Furthermore, the positions of the ZnO diffraction peaks remained essentially unchanged, while their intensities increased with higher ZnO loading, confirming that the crystal structure of ZnO was preserved in the composite films.

**Figure 4:**
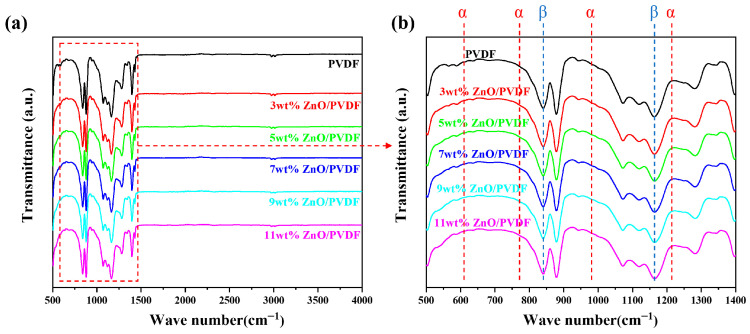
FTIR patterns of ZnO/PVDF-TrFE film. (**a**) Wavenumber range of 200–4000 cm^−1^; (**b**) magnified view in the wavenumber range of 500–1400 cm^−1^.

The FTIR spectra of ZnO/PVDF-TrFE composite films with varying ZnO doping contents are shown in Figure 4. As reported by Jia [30], the characteristic absorption peaks of the α-phase are located at 614 cm^−1^, 764 cm^−1^, 976 cm^−1^, and 1234 cm^−1^, while those of the β-phase are observed at 840 cm^−1^ and 1175 cm^−1^ [31]. From Figure 4b, it can be seen that the intensities of the absorption peaks at 840 cm^−1^ and 1175 cm^−1^ in the ZnO/PVDF-TrFE composite films gradually increase with higher ZnO content. This indicates that the proportion of the β-phase in the composite films rise with increasing ZnO loading.

To quantitatively characterize the content of the polar β-phase (*F_β_*) in ZnO/PVDF-TrFE composite films, the Beer-Lambert law [32,33] can be employed for calculation:

(1) $$F_{\beta }=\frac{A_{\beta }}{\frac{K_{\beta }}{K_{\alpha }}A_{\alpha }+A_{\beta }}=\frac{A_{\beta }}{1.26A_{\alpha }+A_{\beta }}$$

where *A_β_* and *A_α_* are the absorption intensities of the infrared peaks at 840 cm^−1^ and 764 cm^−1^ in the FTIR spectrum, respectively. The absorption coefficients *K_β_* and *K_α_* are constants with values of 7.7 × 10^4^ and 6.1 × 10^4^, respectively.

Based on the calculations, the *F_β_* values for pure PVDF-TrFE, 3 wt% ZnO/PVDF-TrFE, 5 wt% ZnO/PVDF-TrFE, 7 wt% ZnO/PVDF-TrFE, 9 wt% ZnO/PVDF-TrFE, and 11 wt% ZnO/PVDF-TrFE were 20.44%, 20.66%, 23.43%, 23.55%, 24.76%, and 23.50%, respectively (As shown in Table 1). It can be observed that with the increase in absorption peak intensity, the β-phase proportion also rises within the ZnO doping range from 3 wt% to 9 wt%. However, the β-phase content in 11 wt% ZnO/PVDF-TrFE is lower than that in the 9 wt% sample. A possible explanation is that, with increasing ZnO content, partial agglomeration of ZnO nanoparticles occurs, which reduces their contact area with PVDF. This decrease in interfacial interaction between ZnO and the -CH_2_ dipoles may inhibit crystallization and ultimately affect the β-phase content in the composite film.

**Figure 5:**
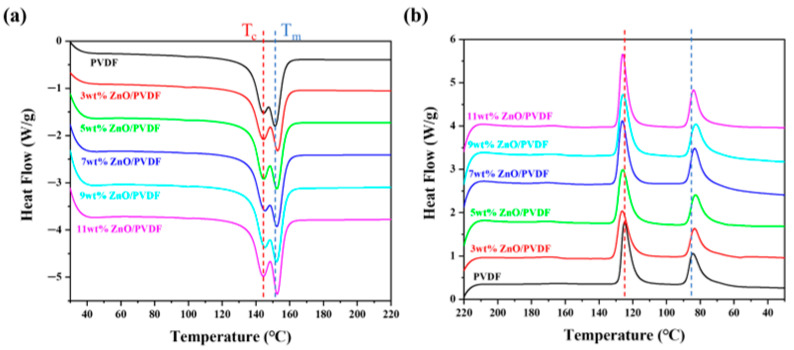
(**a**) Melting and (**b**) crystallization behaviors of the ZnO/PVDF-TrFE composite films.

The melting behavior (a) and crystallization behavior (b) of the ZnO/PVDF-TrFE composite films are shown in Figure 5. In Figure 5a, T_c_ denotes the Curie temperature above which the piezoelectric film loses its piezoelectricity as the material transitions from a ferroelectric to a paraelectric phase, resulting in the disordering of internal dipoles, while T_m_ represents the melting temperature beyond which the polymer enters a viscous flow state.

When the ZnO mass ratio ranges from 3 wt% to 11 wt%, the melting temperature of the composite is higher than that of pure PVDF-TrFE, indicating that the addition of ZnO enhances the crystallinity of the material. Combined with the increased intensities of the diffraction peaks at 20.3° and 35.8° in Figure 3 as the ZnO content rises, this further confirms that ZnO indeed acts as a nucleating agent during the crystallization process and promotes the crystallization of PVDF-TrFE. Figure 5b shows the crystallization behavior of samples with varying ZnO doping levels after heating. As can be observed, all six samples exhibit two peaks around 125 °C and 86 °C, which correspond to the crystallization temperature and the paraelectric-to-ferroelectric phase-transition temperature, respectively. The crystallization temperatures of the ZnO/PVDF-TrFE composite films are consistently higher than that of pure PVDF-TrFE (crystalline regions generally exhibit higher melting points than amorphous ones). Combined with the relevant findings from FTIR, XRD, and morphological analyses, these results strongly demonstrate that the incorporation of nano-ZnO promotes the β-phase crystallization of PVDF-TrFE.

### 3.2. Piezoelectric Properties of the ZnO/PVDF-TrFE Composite Films

**Figure 6:**
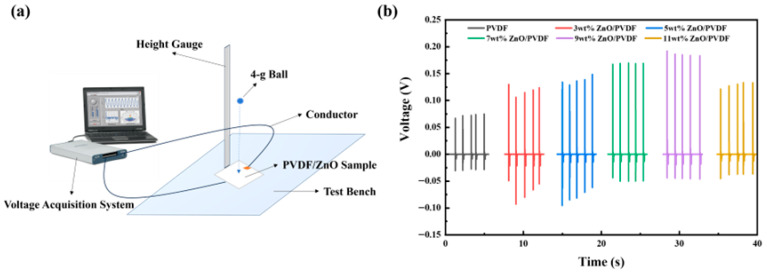
(**a**) Electrical characterization platform for ZnO/PVDF-TrFE composite films, (**b**) Output electrical signals of ZnO/PVDF-TrFE composite films with different ZnO doping levels.

This study established an electrical characterization platform to comprehensively evaluate the electrical performance of ZnO/PVDF-TrFE devices and investigate the influence of various factors on their performance. Figure 6a illustrates the working principle of the platform: a 4 g sphere is released from a fixed height to impact the surface of the ZnO/PVDF-TrFE device (5 cm × 5 cm). Upon sensing the external impact force, the ZnO/PVDF-TrFE device generates an output voltage signal. A voltage acquisition card is connected to the device via leads to capture the voltage signal, which is then recorded and analyzed using a computer equipped with DAQ-Express software (Version 5.1).

Figure 6b records the output electrical signals of ZnO/PVDF-TrFE composite films with different ZnO doping levels. The output voltage signal of the ZnO/PVDF-TrFE composite film increases with higher ZnO content, reaching a maximum at 9 wt% ZnO doping. This observation is consistent with the XRD characterization results, which showed that the 9 wt% ZnO/PVDF-TrFE sample had the highest *F_β_* value. When the ZnO doping level is increased to 11 wt%, the output voltage signal of the composite decreases. A possible explanation for this trend is that the introduction of ZnO increases the steric hindrance of the C–F dipoles. Excessive ZnO doping leads to overly pronounced steric effects, which significantly hinders the formation of β-phase crystals, thereby reducing the β-phase content in the copolymer and consequently lowering the output voltage signal of the ZnO/PVDF-TrFE composite film.

In summary, based on the analysis of the output voltage signal values, the composite device prepared with a 9 wt% ZnO doping content exhibits the optimal performance among the investigated samples. Therefore, 9 wt% ZnO/PVDF-TrFE composite film device is selected as the optimal candidate for subsequent research work.

### 3.3. Ultrasonic Transmission and Reception Performance of ZnO/PVDF-TrFE Piezoelectric Composite Film

Under excitation by a high-frequency signal, the piezoelectric film undergoes periodic high-frequency vibration and emits ultrasonic waves. When these ultrasonic waves encounter interfaces with different acoustic impedances (such as air or human skin), they exhibit distinct interfacial behaviors depending on the degree of impedance matching with PVDF. For instance, in air, since the acoustic impedance of air is 413 rayl while that of PVDF is 4 Mrayl, the significant impedance mismatch causes the ultrasonic waves to be almost completely reflected at the interface, resulting in negligible energy loss. However, at the surface of human skin, with an acoustic impedance of approximately 1.5 Mrayl, partial transmission of ultrasonic waves occurs at the skin interface, leading to a certain degree of energy loss [34,35,36,37].

**Figure 7:**
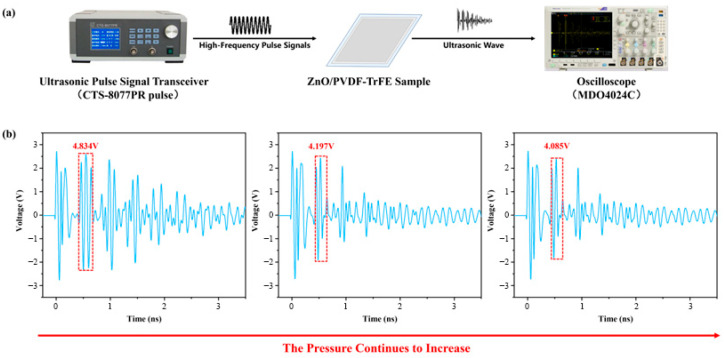
(**a**) Ultrasonic response characterization platform for the ZnO/PVDF-TrFE composite film device, (**b**) Ultrasonic echo signals of the ZnO/PVDF-TrFE composite film device under different levels of sustained static pressure.

As shown in Figure 7a, during the testing process, an ultrasonic pulse transmitter/receiver (CTS-8077PR) applies a high-frequency pulse signal to the 9 wt% ZnO/PVDF-TrFE composite film device, exciting the film to undergo microscopic vibration and thereby emit ultrasonic waves (at the same frequency as the excitation signal). After reflection and transmission at the interface, the reflected ultrasonic waves are amplified by the ultrasonic pulse transmitter/receiver and displayed in real-time on the connected oscilloscope (MDO4024C).

By maintaining a constant contact area between the finger and the sensor while gradually increasing the contact pressure, we investigated the relationship between the intensity of the ultrasonic echo signal and the contact pressure. Under a fixed contact area, the static and sustained pressure on the effective sensing region was progressively raised. As shown in Figure 7b, the intensity of the ultrasonic echo signal collected from the contact interface gradually decreased with increasing local pressure. This is because the elevated static pressure leads to an increase in the local density of the skin, which in turn raises its acoustic impedance within the contact region. As the acoustic impedance of the skin (≈1.5 Mrayl) approaches that of PVDF (≈4 Mrayl), the impedance mismatch between the two materials is reduced. Consequently, a larger portion of the ultrasonic energy is transmitted into the skin tissue, resulting in greater energy loss and a corresponding decrease in the reflected echo signal.

The ultrasonic signal results demonstrate that our device is capable of detecting subtle fluctuations in static force. When the sensor is applied to wrist pulse monitoring, this static force information can be used to evaluate whether the device is properly attached to the wrist.

### 3.4. Applications of ZnO/PVDF-TrFE Composite Film Device in Wearable Hand Pulse Monitoring

**Figure 8:**
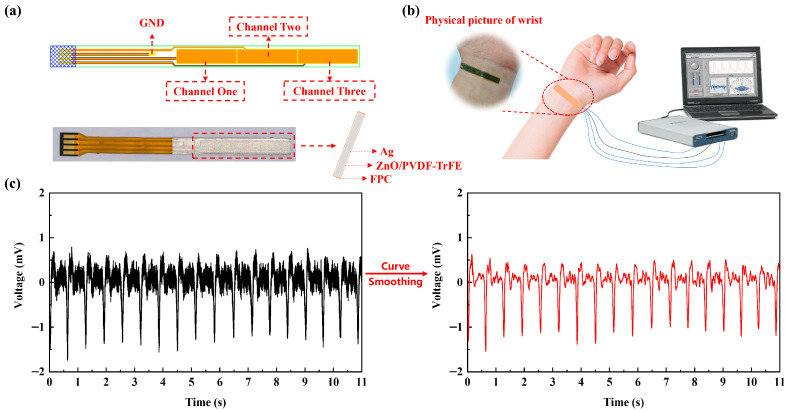
(**a**) ZnO/PVDF-TrFE composite film device based on a three-channel FPC substrate, (**b**) Experimental setup for pulse signal detection, (**c**) Pulse signal acquired and identified by the ZnO/PVDF-TrFE composite film device.

To better capture wrist pulse signals, a three-channel flexible conductive substrate was designed, as shown in Figure 8a. During wearable use, positional misalignment may occur between the pulse location and the sensing device on the flexible substrate. The multi-channel design helps prevent loss of contact due to such relative displacement, enabling faster and more accurate pulse signal detection and ensuring stable device operation. Additionally, the multi-channel design provides redundancy and fault tolerance. While damage to a single-channel electrode can easily lead to system failure, the multi-channel configuration allows for immediate substitution with backup channels, significantly enhancing the device’s operational reliability and continuity. Figure 8b presents the experimental setup used to validate the pulse-sensing capability of the ZnO/PVDF-TrFE composite film device in the laboratory. The device is placed in contact with the wrist pulse point, and the acquired pulse signals are displayed via a data acquisition card and a computer. Figure 8c displays the pulse signal measured from the test subject. The signal is consistent with that obtained using conventional reference equipment, indicating the high accuracy of pulse detection achieved by the ZnO/PVDF-TrFE composite film device. By applying curve-smoothing processing, artifacts and other interference present in the raw test results can be removed, allowing clearer visualization of the wrist pulse waveform. Based on a short-duration test (approximately 10 s), the heart rate of the subject was analyzed and determined to be 96 beats per minute.

## 4. Conclusions

In this study, ZnO/PVDF-TrFE composite films with varying ZnO doping levels (3–11 wt%) were successfully fabricated and systematically characterized. Through structural, thermal, and electrical performance analyses, the regulatory mechanism of ZnO on the crystallization behavior and piezoelectric response of the material was clarified, and its potential for flexible sensing and biosignal detection was verified. XRD and FTIR analyses demonstrated that the incorporation of ZnO significantly promotes the formation of the polar β-phase in PVDF-TrFE. At a ZnO content of 9 wt%, the β-phase content (*F_β_*) reached a maximum of 24.76%, corresponding to optimal piezoelectric performance. Devices based on the optimal 9 wt% formulation exhibited stable ultrasonic transmission and reception capabilities under high-frequency pulse excitation. Experiments confirmed that the device can sensitively detect minor static pressure fluctuations (e.g., contact pressure) through changes in ultrasonic echo signal intensity, providing a feasible solution for wearable fit monitoring. Furthermore, a sensor designed with a three-channel flexible substrate accurately captured human wrist pulse signals, demonstrating the practicality and reliability of the device in flexible bioelectronics.

## Figures and Tables

**Table 1:** The absorption intensities of ZnO/PVDF-TrFE at 764.16 cm^−1^ and 839.85 cm^−1^ and the calculated *F_β_* results.

Wave Number	Transmittance (a.u.)					
	PVDF	3 wt%ZnO/PVDF	5 wt%ZnO/PVDF	7 wt%ZnO/PVDF	9 wt%ZnO/PVDF	11 wt%ZnO/PVDF
764.16 cm^−1^	87.10	87.27	89.00	89.03	89.30	88.47
839.85 cm^−1^	28.20	28.62	34.31	34.55	37.02	34.25
*F_β_*	20.44%	20.66%	23.43%	23.55%	24.76%	23.50%

## Data Availability

The original contributions presented in this study are included in the article/Supplementary Materials. Further inquiries can be directed to the corresponding author.

## Supplementary Materials

The following supporting information can be downloaded at: https://www.mdpi.com/article/10.3390/bios16040187/s1, Figure S1: SEM and ESM-EDS images of pure PVDF-TrFE composite film.
