# Supplementary material for: Enhancement of Piezoelectric Performance in PVDF via ZnO Doping and Its Application in Wearable Real-Time Monitoring of Human Radial Pulse
Source: Biosensors (Basel). 2026 Mar 24;16(4):187. doi: 10.3390/bios16040187 (PMC13114083; doi:10.3390/bios16040187)
Supplement: Supplementary file 1 [file biosensors-16-00187-s001.zip › biosensors-4182647-supplementary.pdf]

biosensors

MDPI

Supplementary Information

Enhancement of Piezoelectric Performance in PVDF via ZnO Doping and Its Application in Wearable Real-Time Monitoring of Human Radial Pulse

Hao Zhu [1], Xiang Guo [2], Qiang Liu [3] and Qian Zhang [1,*]

[1] School of Integrated Circuit Science and Engineering, University of Electronic Science and Technology of China, Chengdu 611731, China; haozhu@jsatec.com
[2] Mianyang Municipal Health Commission, Mianyang 621000, China; swjw301@163.com
[3] School of Materials and Energy, University of Electronic Science and Technology of China, Chengdu 611731, China; qiangrye@163.com
* Correspondence: zq@uestc.edu.cn

Figure S1 presents the SEM and SEM-EDS images of the pure PVDF-TrFE film. As shown, the cross-sectional structure of the pure PVDF-TrFE film is dense and its surface is smooth, with no obvious pores, cracks, or crystalline phase-transition regions observed. The SEM-EDS elemental mapping reveals that C and F are uniformly distributed throughout the film without local enrichment or depletion, indicating a homogeneous material composition, absence of phase separation, and no detectable contamination from other impurity elements. The structural and compositional uniformity of the pure PVDF-TrFE film provides a reliable reference for the construction of the ZnO/PVDF-TrFE composite system. Furthermore, the incorporation of ZnO achieves uniform dispersion of the functional filler and effective interfacial bonding while maintaining the structural integrity of the matrix, which offers crucial morphological and structural evidence for the enhanced piezoelectric and dielectric performance of the ZnO/PVDF-TrFE composite material [26,27].

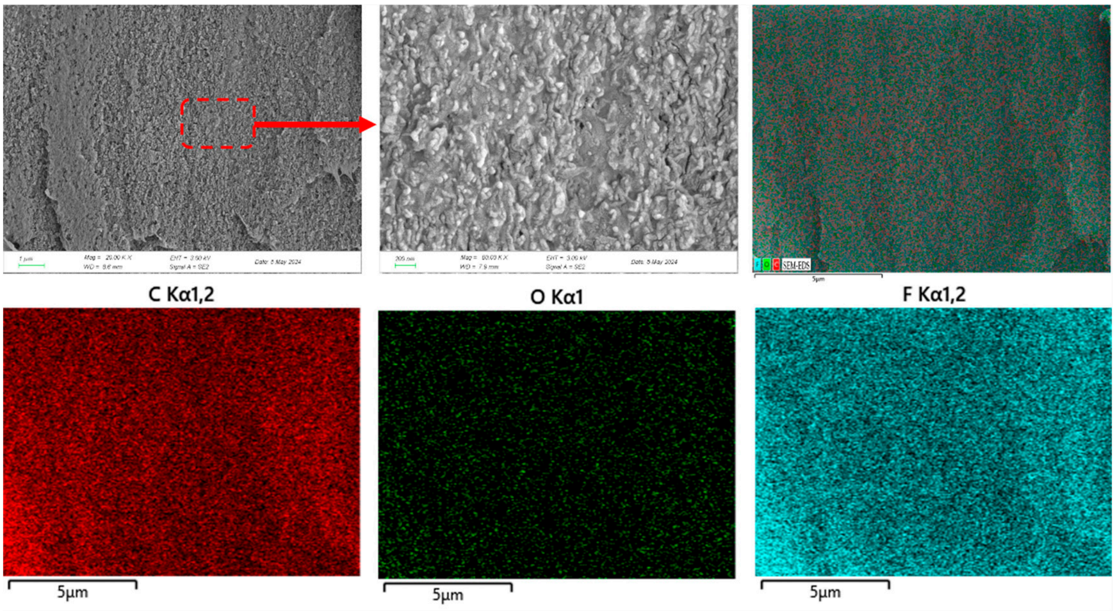

Figure S1. SEM and ESM-EDS images of pure PVDF-TrFE composite film.

 https://doi.org/10.3390/bios16040187

References

26 Gregorio Jr, R. Determination of the α, β, and γ crystalline phases of poly(vinylidene fluoride) films prepared at different conditions. J. Appl. Polym. Sci. **2006**, 100, 3272–3279.

27 Esterly, D.M.; Love, B.J. Phase transformation to β-poly(vinylidene fluoride) by milling. J. Polym. Sci. Part B Polym. Phys. **2004**, 42, 91–97.

Disclaimer/Publisher's Note: The statements, opinions and data contained in all publications are solely those of the individual author(s) and contributor(s) and not of MDPI and/or the editor(s). MDPI and/or the editor(s) disclaim responsibility for any injury to people or property resulting from any ideas, methods, instructions or products referred to in the content.

https://doi.org/10.3390/bios16040187
